# Diabetes: Is There a Future for Pharmacogenomics Guided Treatment?

**DOI:** 10.1002/cpt.1484

**Published:** 2019-07-12

**Authors:** Ewan R. Pearson

**Affiliations:** ^1^ Division of Population Health & Genomics School of Medicine University of Dundee Dundee UK

## Abstract

Diabetes is a disease defined on the basis of hyperglycemia. There are monogenic forms of diabetes where defining the genetic cause has a dramatic impact on treatment—with patients being able to transition from insulin to sulfonylureas. However, the majority of diabetes is type 2 diabetes. This review outlines the robust evidence accrued to date for pharmacogenetics of metformin, sulfonylureas, thiazolidinediones, and dipeptidyl peptidase‐4 inhibitors but highlights that these variants will only be of clinical utility when the genotype is already known at the point of prescribing. The future of pharmacogenetics in diabetes and other common complex disease relies on a paradigm shift—that of preemptive panel genotyping and use of clinical decision support tools to assimilate this genetic information with other clinical phenotypic data and to present this information simply to the prescriber. Given the recent dramatic fall in genotyping costs, this future is not far off.

To date, clinically actionable pharmacogenomics has largely been limited to severe idiosyncratic adverse drug reactions, to variation in drug metabolism, and to treatment outcome in cancer chemotherapeutics where the somatic mutations drive the choice of targeted intervention. In this review, I will focus on pharmacogenomics in type 2 diabetes and use this not only to outline the recent advances in the field but to address the challenges that are faced when considering genetics of treatment efficacy and (usually mild–moderate) side effects in common complex disease.

## The Complex Etiology of Diabetes

Diabetes, like many complex diseases, is diagnosed based upon a threshold being achieved in a normally distributed trait. In the case of diabetes, this is a fasting blood glucose >7 mmol/L with the threshold level being that at which the risk of microvascular complications emerges. There are many reasons why an individual's blood glucose can rise above this threshold; however, traditionally two groups are identified—type 1 and type 2 diabetes. Whilst type 1 diabetes has a clear etiology (autoimmune islet beta‐cell destruction), type 2 diabetes is essentially a diagnosis of exclusion—it is what is left after all other known causes are ruled out. Thus, it seems likely that within this categorical bin that is type 2 diabetes there may well be definable subgroups of distinct etiology. Over the last 20 years, an increasing number of monogenic forms of diabetes have been identified, which are often still mistaken for type 2 diabetes. As will be outlined later, these monogenic forms of diabetes can have extreme response to targeted diabetes treatments and represent robust exemplars for pharmacogenomics in the diabetes clinic. However, whilst it is tempting to assume that as we gain more knowledge of diabetes etiology we will slice the group with type 2 diabetes into discrete subtypes, we need to recognize that true type 2 diabetes is indeed a polygenic disease. Recent genetic studies have established that there are likely thousands of common risk variants that contribute to type 2 diabetes risk, and the contribution of rare or low frequency variants, whilst individually of large effect, do not contribute greatly to the overall prevalence of type 2 diabetes.^1^

## Treatment Response in Type 2 Diabetes

**Figure 1:**
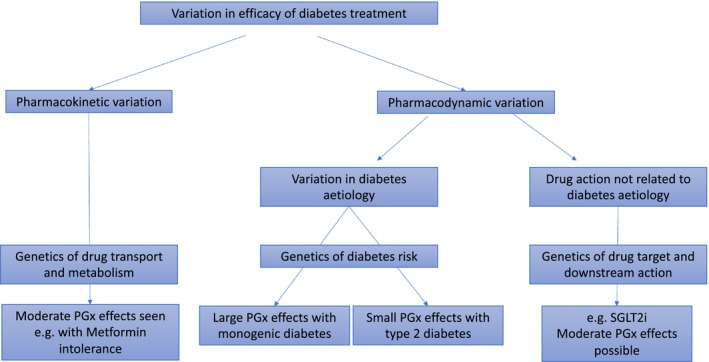
A framework for evaluating PGx in type 2 diabetes. PGx, pharmacogenetics; SGLT2i, sodium glucose transporter 2 inhibitor.

Traditionally pharmacogenomics of drug efficacy is divided into variants that alter drug pharmacokinetics (PK), and variants that alter drug pharmacodynamics (PD) (**Figure** **1**). In the context of a complex disease such as type 2 diabetes where there is considerable etiological variation, it is helpful to divide PD variation into differences in drug response that reflect the underlying etiological variation and differences that do not. Most diabetes drugs act to attempt to reverse the pathophysiological (etiological) defects that contribute to the development of diabetes and thus differences in etiology (e.g., beta‐cell failure) are likely to impact on response to drugs that primarily work on this pathway (e.g., sulfonylureas). As such we would anticipate that understanding the genetic etiology of diabetes should translate to pharmacogenomics of that drug/etiological pathway. Conversely, as will be described later for metformin, understanding genetic architecture of diabetes drug response may provide insights into diabetes etiology. By contrast, some drugs (e.g., sodium glucose transporter 2 inhibitors) lower blood glucose via a distinct mechanism (in this case inhibiting glucose reabsorption resulting in glycosuria) that is not etiological for diabetes, and therefore in this scenario, genetic variation in etiology is unlikely to map to variation in drug response.

## Small Pharmacogenetic Effects are Actionable if Genotype is Free and Available at the Point of Prescribing

Diabetes drugs vary in their efficacy by class. Metformin and sulfonylureas are probably the most potent, with hemoglobin A1c (HBA1c) reduction of 1–1.5% often seen with dipeptidyl peptidase‐4 (DPP‐4) inhibitors achieving a mean HbA1c reduction of ~ 0.6%. At this point, for the nondiabetes audience, it should be noted that glycemia is measured as an absolute measure of percentage‐glycated hemoglobin. Thus, absolute change in HbA1c can be mistaken to be a relative change—a 1% improvement in HbA1c is a robust effect, not a trivial effect, and is associated with a ~ 30–40% reduction in risk of microvascular complications. In clinical trials a difference between interventions of 0.3% is considered to be a clinically important difference.

Given that pharmacogenetic effects of diabetes drug treatment are likely to reflect the underlying etiology (**Figure** **1**) and that true type 2 diabetes is highly polygenic, consisting of multiple small‐effect etiological variants, it is likely that for treatment efficacy the pharmacogenetic effects will be small. We have shown this to be the case for metformin response, where the heritability is spread evenly across all the chromosomes, rather than being driven by a few loci of large effect.^2^ The exception to this are the large effects seen in monogenic diabetes or the modest effects seen for drug intolerance.

The traditional approach to pharmacogenomics is to request a genetic test before issuing a prescription. This works where the risk of harm is high (e.g., severe adverse drug reaction) or the risk of nonefficacy is high (e.g., chemotherapy) compounded by the high cost of the drug. Yet in type 2 diabetes, where pharmacogenetic effects are likely to be small and drug costs are also relatively small, a clinician or healthcare provider is unlikely to sanction the cost of genotyping/sequencing and the time taken to get the result before deciding on diabetes drug choice. However, the cost of sequencing and genotype arrays is falling dramatically. Now it is possible to undertake genome‐wide genotyping, with high‐density imputation for ~ £30/$40. Most but not all absorption, distribution, metabolism, excretion (ADME) content is captured as would be other variants associated with drug efficacy. At this price, preemptive genotyping with actionable genotypes embedded in the medical record has the potential to be a reality. With appropriate information technology infrastructure and mapping of genotype to a clear clinical action, the genotyping information would be free and instantly available at the point of prescribing, embedded in a clinical decision support tool. At this point the genotype will become as easy to use to guide decisions as body mass index or sex. At this point, small to modest pharmacogenetic effects can be used to guide prescribing—if a decision between two diabetes treatments is in equipoise, the genetic information can help steer the decision.

This paradigm shift represents a challenge for healthcare providers and payers. The current approach to implementation of new treatments or diagnostics requires evidence of efficacy and cost effectiveness, often in the form of a clinical trial. Yet for pharmacogenetics the potential combinations of drug–gene interactions are many, and it would be unfeasible for every combination to be tested in an outcome trial. It is more realistic to ensure that as long as there is a sufficient level of evidence to support the introduction of pharmacogenetic‐guided prescribing, there is a rigorous evaluation of outcomes to enable a full assessment of effectiveness and cost‐effectiveness *post hoc*.

## Pharmacogenomics in Common Complex Disease—Probabilistic Rather THAN Definitive Outcomes

**Figure 2:**
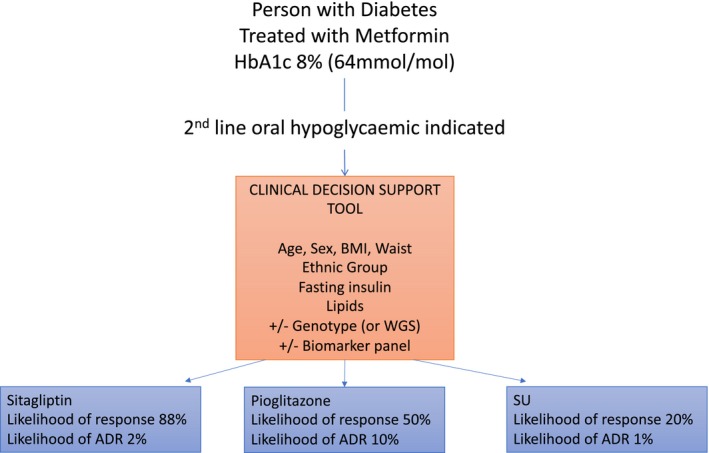
The future of pharmacogenetics in type 2 diabetes—incorporating genotype into a clinical decision support tool. A primary care physician starts to prescribe a new treatment for a patient whose glycemic control is above target. A clinical decision support tool built into the prescribing tool queries simple clinical and laboratory measures, actionable genotypes, and other biomarkers. The output is a probability of likely response and likely adverse drug reaction (ADR). The physician can use this to guide a discussion with their patient about the best drug to initiate to maximize benefit and minimize harm. BMI, body mass index; HbA1c, glycated hemoglobin A1c; SU, sulfonylurea; WGS, whole‐genome sequence.

One final concept that applies to pharmacogenomics of common complex diseases like type 2 diabetes is that the genetics are not likely to be used to give a definitive prediction. So, for example, when prescribed abacavir, if an individual has the HLA‐B*5701 genotype, then the evidence is clear—they should not receive abacavir.^3^ In type 2 diabetes this is unlikely to ever be the case. In part this is because the effect size is likely to be small (as already discussed) and, importantly, because the response trait is highly variable within individuals. Thus, whilst on average a group of patients carrying a “good response” allele will respond better than a group who don't carry this allele, at an individual level there will be some who carry the good response allele who respond poorly; similarly there will be individuals who do not carry the good response allele who respond well. Therefore, we cannot predict with any degree of certainty unless the predicted allelic effect is very large (which, other than in monogenic diabetes, it isn't). Therefore, genotype will be used in combination with clinical characteristics to develop a probabilistic response prediction (**Figure** **2**), where, for example, a patient is predicted to have a 73% probability of achieving a target HbA1c with one drug but only a 50% probability with an alternative drug. In this scenario, this information on probability of response can be used by the doctor and patient to decide on what treatment should be prescribed, weighing up the characteristics of the drug, potential side effects, and patient preference.

## Drug Response in Diabetes—the Clinical Phenotype

Before moving to examples of how genetic variation has been established to alter drug response in diabetes, it is important to recognize that genetic variation is only one aspect to be considered in predicting the efficacy of a diabetes drug. Traditionally clinical trials did not consider patient phenotype when evaluating drug response. However, recent studies have revisited these trials and shown that even simple parameters like body mass index and sex matter. For example, obese women respond very well to thiazolidinediones, whereas slim men respond well to sulfonylureas.^4^ With more systematic evaluation of phenotype and incorporating measures of insulin resistance and beta‐cell function, patients with diabetes have been divided into five groups or subtypes.^5^ It is likely that these groups will respond differently to diabetes treatments. In this context, genetic variation will need to be considered layered upon such clinical and physiological variation in order to guide treatment choice.

## Monogenic Diabetes—Pharmacogenomic‐Guided Treatment is Already Part of Clinical Practice

As has been mentioned, monogenic diabetes represents the exception to the concepts outlined above. The term monogenic diabetes covers all forms of diabetes caused by a single‐point mutation or deletion in or of a gene. There are many rare forms of monogenic diabetes that are beyond the scope of this review. Here, I will focus on the monogenic beta‐cell disorders that cause maturity onset diabetes of the young (MODY) and the rare beta‐cell disorders that cause neonatal diabetes, as these genetic subtypes can be treated differently from “normal” type 1 or type 2 diabetes.

MODY is itself a heterogeneous condition with dominant mutations in more than 10 genes found to be causal (for more information see ref. 6^6^). MODY traditionally presents as non–insulin‐requiring diabetes with onset before the age of 25 years, with a three‐generation family history. The most common form of MODY is caused by mutations in the gene, *HNF1A*, encoding the transcription factor hepatocyte nuclear factor 1α. Because HNF1A MODY often presents at a young age it may be mistaken for type 1 diabetes; or, as it can present at a later age without insulin requirement, it can be mistaken for type 2 diabetes. However, unlike type 2 diabetes, this genetically defined subgroup is exquisitely sensitive to sulfonylurea treatment. A randomized crossover trial of metformin and gliclazide (a sulfonylurea) in patients with type 2 diabetes and with HNF1A MODY established that the MODY patients were fivefold more sensitive to the glucose‐lowering effects of sulfonylureas than patients with type 2 diabetes.^7^ This finding led to successful transition of patients (who had been incorrectly diagnosed as having type 1 diabetes) from insulin treatment to low‐dose sulfonylurea, often after many years of being labeled insulin dependent.^8^ The pharmacogenetic effect size here is so large that knowing an individual's genotype will prompt definitive treatment advice—use a low‐dose sulfonylurea. However, the caveat is that as MODY is rare (~ 3% of diabetes diagnosed less than 30 years of age) there is no logic in testing for MODY in every patient thought to have type 2 diabetes before prescribing a sulfonylurea as most won't have MODY, and many patients with type 2 diabetes will respond well to sulfonylureas anyway.

Another form of MODY is caused by a genetic defect in glucokinase, the pancreatic glucose sensor. This presents as mild fasting hyperglycemia present from birth but with relatively normal postprandial glucose rise and HbA1c, and as a result with no long‐term increased risk of microvascular complications.^9^ However, if a child is tested (for another reason) and found to have raised blood glucose, they are often assumed to have type 1 diabetes and started on insulin treatment. Interestingly, insulin treatment makes no difference to the level of HbA1c^10^ and no diabetes treatment is required in people with Glucokinase‐MODY. As a result, patients misdiagnosed as having type 1 diabetes can stop their insulin, and patients misdiagnosed with type 2 diabetes can stop all oral therapy.

Neonatal diabetes (NDM) is a form of diabetes that presents within the first 6 months of age. At this age, it is very difficult to acquire an autoimmune type 1 diabetes and highly likely that there is a genetic etiology. Fifty percent of cases of NDM have been found to be caused by heterozygous mutations in the genes encoding the K_ATP_ channel subunits KIR6.2^11^ and SUR1.^12^ These mutations result in the K_ATP_ channel being insensitive to intracellular adenosine triphosphatase (ATP) and therefore remaining open, holding the beta cell in a hyperpolarized state, and unresponsive to glucose.^11^ The K_ATP_ channel is the site of action of sulfonylureas, which close the channel and promote insulin secretion. In these patients, sulfonylureas used at high dose (2–4 times the maximum adult dose/kg) are able to close the mutant K_ATP_ channels and stimulate insulin secretion.^11^ This work led to the successful transition off lifelong insulin treatment onto sulfonylurea treatment in 90% of patients with NDM.^13^ Another striking example of pharmacogenomics.

## Pharmacogenomics in Type 2 Diabetes

There are many studies reporting genetic impact on diabetes drug response in type 2 diabetes. These are often studies done in small numbers (e.g., 100 patients) and are not replicated. As the anticipated effect sizes are small in type 2 diabetes, it is likely that most of the reports in the literature are false positives. In this review I do not attempt to compile all the pharmacogenetic studies of diabetes drugs because there are a number of recent reviews that have done this.^14^, ^15^, ^16^ Instead I will focus on two key areas—pharmacogenetic effects that are robust (on the basis of replication and/or biological plausibility) and have the potential to translate into clinical care, and how pharmacogenetics can be used to provide insight into diabetes etiology and drug action, specifically in relation to metformin. I will focus on metformin, sulfonylureas, thiazolidinediones, and DPP‐4 inhibitors. There are only limited pharmacogenetic studies published for sodium glucose transporter 2 inhibitors and glucagon‐like peptide‐1 receptor agonists.

## Metformin Efficacy

Metformin is the first‐line drug treatment for type 2 diabetes. It is derived from the French lilac and has been in clinical use for over 60 years. Yet its mechanism of action remains much debated—for an overview of its action see ref. 17.^17^ Metformin is a highly effective treatment that is associated with weight loss and probable cardioprotection, and it is being investigated for its potentially beneficial effects on cancer risk and outcomes^18^ and aging.^19^ It should be noted, however, that metformin also causes quite considerable gastrointestinal (GI) side effects in ~ 10% of individuals who are prescribed the drug, leading to cessation in ~ 5%. The mechanisms for GI intolerance are also poorly understood. The considerable benefits with confusion about mechanism of action and side effects makes this an intriguing drug worth studying!

In a novel approach to investigate metformin mechanism, a genome‐wide association study (GWAS) was undertaken in patients with type 2 diabetes treated with metformin, with HbA1c reduction after initiation as the outcome. In the first study reported in 2011,^20^ the initial discovery GWAS was ~ 1,000 patients, with a total sample size of 4,200 patients. Here, we identified that a locus on chromosome 11 that includes the genes NPAT and ATM was associated with glycemic response to metformin. Subsequently, the Metformin Genetics Consortium (https://www.pgrn.org/metgen.html) was formed, and the available sample size was increased to 12,910 patients, with the discovery that an intronic variant altering SLC2A2 (GLUT2) expression levels was associated with glycemic response to metformin.^21^ These two metformin loci (*NPAT*/*ATM* and *SLC2A2*) are the most robustly replicated pharmacogenetic variants in type 2 diabetes. As with many genetic studies, they raise more questions than they answer. For the *NPAT*/*ATM* locus it is tricky to be sure of the causal variant and gene, and work is ongoing in mouse models to investigate the mechanism, but it is tempting to conclude that the cancer gene, *ATM*, is causal. Patients who carry recessive mutations in *ATM* develop ataxia telangiectasia, which is a condition characterized by ataxia, lymphoproliferative cancer, immunoglobulin deficiency, *and* diabetes and insulin resistance.^22^ *SLC2A2* is a more plausible gene where variation can alter glycemic response to metformin, as this is the main glucose transporter in the liver and is involved in glucose transport in the gut, beta cell, and hypothalamus. The *SLC2A2* single‐nucleotide polymorphism rs8192675 is the top expression quantitative trait locus for GLUT2 in the liver, with the increased efficacy allele being associated with lower expression of GLUT2.^21^ The mechanism for how reduced expression of GLUT2 alters metformin action, and whether this relates to a liver effect or gut effect remains under investigation. The effect size for rs8192675 is not small: In obese individuals, there is a 0.33% greater HbA1c reduction in the 9% of white Americans who carry two copies of the C allele compared with those who carry two copies of the T allele.^21^ This is the equivalent of a difference in metformin dose of 550 mg, or over half the effect seen with the initiation of a DPP‐4 inhibitor. In black Americans, the good response C allele is homozygous in 49% of the population, suggesting that this genetic variant will have a large population effect on metformin response in this ethnic group. Will these genetic effects translate into clinical practice? The challenge is that metformin is a cheap, effective drug that is used first‐line, and has benefits beyond glycemia (such as weight lowering and potential reduction in cancer risk), and it is therefore unlikely that it will displaced from its first line‐position, even for those patients where it is likely to be less effective. Studies need to be done to establish whether dose adjustment can compensate for the pharmacogenetic effect, enabling genotype‐based dosing of metformin.

## Metformin Intolerance

Approximately 5–10% of patients cannot tolerate metformin, largely due to intestinal side effects (bloating, abdominal pains, and diarrhea). Metformin is a cation with low lipid solubility, requiring its active transport across cell membranes by a variety of transporters, including the organic cation transporters. In the gut, metformin is predominantly absorbed in the small intestine, with ~ 30% secreted unchanged in the feces. Metformin is highly concentrated in the enterocytes of the jejunum, with concentrations as high as 500 μg/g (30–300 times higher than seen in plasma). Many underlying mechanisms for GI intolerance to metformin have been postulated (reviewed in ref. 23^23^): a direct effect of the high metformin concentration of metformin in the enterocytes where metformin acts as a cellular poison, alteration of the gut microbiome by metformin,^24^, ^25^ alteration of serotonin or histamine uptake or metabolism, a reduction in bile acid reabsorption resulting in increased bile acid exposure to the colon.

**Figure 3:**
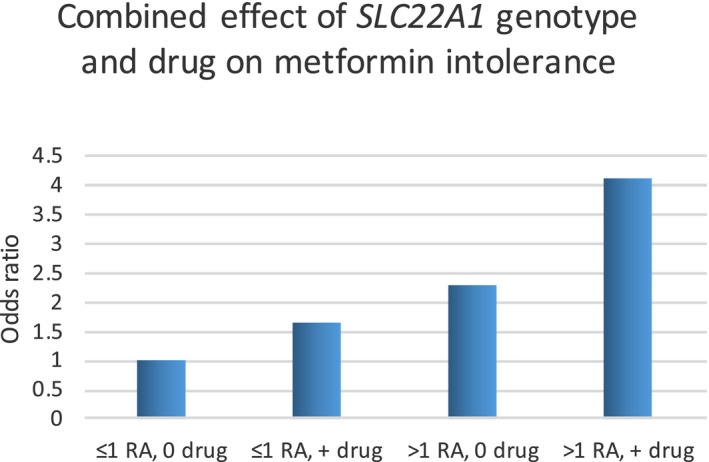
The combined effect of reduced function *SLC22A1* genotypes and organic cation transporter 1 (OCT1) interacting drugs on likelihood of metformin intolerance. The reference is those individuals with 1 or 0 risk alleles (RAs) who are not treated with a potential interacting drug (+ drug). Carriage of more than one RA or the use of interacting drugs are associated with intolerance. There is an additive effect in those with more than one RA who are also treated with interacting drugs having the greatest risk of intolerance.

Genetic studies of metformin intolerance have focused on the transport of metformin from the lumen into the enterocytes. The three main transporters for metformin expressed in human gut are organic cation transporter 1 (OCT1, encoded by *SLC22A1*), plasma membrane monoamine transporter (PMAT, encoded by *SLC29A4*), and serotonin transporter (encoded by *SLC6A4*). A recent study in mice suggests OCT3 (encoded by *SLC22A3*) may also play a role,^26^ but this has not yet been established in humans. Variants involving all three known human transporters have now been associated with increased intolerance to metformin. Initially, using the GoDARTS study, Dujic *et al*.^27^ identified that carriers of reduced function variants in *SLC22A1* (encoding OCT1) had increased risk of GI intolerance. GI intolerance in these studies is defined using a proxy phenotype of those who stop metformin within 6 months and switch to an alternative treatment. **Figure** **3** shows that those with two reduced function *SLC22A1* alleles had a more than twofold increase in the odds for metformin intolerance (*P* < 0.001); this effect increased to over fourfold when patients were coprescribed drugs known to inhibit OCT1, such as omeprazole, tricyclic antidepressants, doxazosin, and verapamil.^27^ Dujic followed up with a study of the serotonin receptor, showing that the low‐expressing S* variant in *SLC6A4* was associated with increased intolerance (odds ratio (OR) 1.31, *P* = 0.031), and there was an interaction between *SLC6A4* genotype and *SLC22A1* genotype, such that in patients with two reduced‐function *SLC22A1,* variants, the L* alleles of *SLC6A4*, were associated with a ninefold increase risk of intolerance (OR 9.25, *P* < 10^−4^).^28^ Finally, recently Dawed *et al*. investigated metformin intolerance in the IMI‐DIRECT consortium, where metformin intolerance was self‐reported by patients, resulting in cessation of metformin or the inability to increase the dose beyond 1 g. This study investigated the third gut metformin transporter PMAT and established that the G‐allele at rs3889348, associated with reduced PMAT expression in the gut, was associated with a 1.34 (*P* = 0.005) increased odds of GI intolerance (https://doi.org/10.1101/436980). When considered in the context of the *SLC22A1* reduced‐function variants and the described quantitative trait locus for PMAT in the gut, the odds of intolerance were 2.15 (95% confidence interval (CI), 1.2–4.12) in those carrying three or more risk alleles for these two genes. Given the likely apical location of these transporters, intolerance is associated with reduced uptake into the enterocytes and therefore likely increased luminal metformin concentration in the gut. This suggests that metformin‐induced intolerance is mediated via mechanisms mediated from within the lumen, such as its impact on the microbiome, bile acids, or biogenic amines, rather than increased metformin concentration in the enterocytes.

## Sulfonylurea Efficacy and Hypoglycemia

Sulfonylureas have been in clinical use for as long as metformin, yet unlike metformin, the main mechanism of action is well elucidated. Sulfonylureas bind to the SUR moieties of the K_ATP_ channel, bringing about channel closure, membrane depolarization, and influx of calcium via voltage gated calcium channels, resulting in insulin secretion. Interestingly, despite this pathway being well characterized, there has been limited success in identifying candidate gene variants that alter glycemic response to sulfonylureas. The replicated findings are for variants in *ABCC8*/*KCNJ11* that alters K_ATP_ channel function and for the diabetes risk variant in *TCF7L2*.

The E23K (rs5219) variant in *KCNJ11* and S1369A (rs 757110) variant in *ABCC8* are tightly linked, with the K23/A1369 haplotype being associated with risk for type 2 diabetes. Sulfonylureas differ in the way they interact with the K_ATP_ channel, and functional studies have established that this haplotype has a striking effect on efficacy of the different sulfonylureas.^29^ Here, the K_ATP_ channels containing the K23/A1369 haplotype were more sensitive to inhibition (closure) by gliclazide but less sensitive to closure by tolbutamide, chlorpropamide, and glimepiride. The K23/A1369 haplotype had no impact on glipizide or glibenclamide action. This functional work translates, in part, to that observed in clinical studies. Three Chinese studies report that the K23/A1369 haplotype was associated with greater response to gliclazide.^30^, ^31^, ^32^ The largest of these was a prospective study of 1,268 patients with type 2 diabetes, treated with gliclazide for 8 weeks. Those homozygous for the K23/A1369 haplotype had a small but significantly greater reduction in fasting glucose, 2‐hour glucose, and HbA1c compared with those with the E23/S1369 haplotype.^31^ Other studies do not show an association of the K23/A1369 haplotype with sulfonylurea response, potentially reflecting lack of power of the different sulfonylureas used. For example, the UKPDS study (where chlorpropamide and glibenclamide were the two sulfonylureas used) showed no impact of this haplotype on fasting plasma glucose change at 1 year in 363 patients randomized to sulfonylurea.^33^ This could be explained by the predicted lack of genotype effect on glibenclamide response masking any reduced efficacy predicted for chlorpropamide response.

The diabetes risk variant rs7903146, an intronic single‐nucleotide polymorphism within the TCF7L2 gene has the largest genetic effect on population risk for diabetes to date.^34^ This risk variant is associated with reduced beta‐cell function and is thus a plausible candidate for altering glycemic response to sulfonylureas. In the population‐based GoDARTS study, patients with type 2 diabetes homozygous for rs1225372 (in close linkage disequilibrium with rs7903146) were more likely not to achieve a treatment HbA1c < 7%, (OR 2.16 (1.121–3.86), *P* = 0.009) than patients with no risk variant. This result has been replicated in two independent studies.^35^, ^36^ A recent study, SUGAR‐MGH, evaluated the effect of the diabetes risk variant rs7903146 on response to single‐dose glipizide and 2 days of metformin.^37^ They show that the risk variant is associated with *increased* glucose lowering with glipizide and *increased* glucose lowering with metformin. Whether this reflects acute vs. chronic dosing or biases in the observational data previously reported, there remains some uncertainty over the impact of *TCF7L2* variation and treatment response to sulfonylureas.

The largest effect on sulfonylurea response reported to date relates to genetic variation in the PK of sulfonylureas, rather than the pharmacodynamics. Sulfonylureas are metabolized to an inactive form, in large part by cytochrome P450 (CYP) 2C9. Functional and PK studies have established that the *2 and *3 alleles are associated with reduced function of CYP2C9 and reduced clearance of glibenclamide,^38^ tolbutamide,^39^ glimepiride,^40^ and gliclazide MR.^41^ In 1,073 incident users of sulfonylureas (>90% gliclazide) in the GoDARTS study, the 8% of the population carrying two reduced function alleles at *CYP2C9* (*2 or *3) were 3.4‐fold more likely to achieve therapeutic target than those carrying two normal function alleles; this equates to a 0.5% difference in HbA1c. Similar increased sulfonylurea efficacy was seen for tolbutamide in the Rotterdam study,^42^ where lower tolbutamide dosing was required for similar efficacy in those carrying reduced‐function *CYP2C9* alleles. These large effects would certainly suggest that, if a patient's genotype was known, an altered drug initiation dosing may be beneficial to achieve similar efficacy at lower doses and thus reduce hypoglycemia.

Recently, a further analysis of the GoDARTS study has highlighted the role of P450 oxidoreductase (POR), which transfers electrons from NADPH to the CYP450 enzymes and is essential for normal function of CYP450s.^43^ In this study, sulfonylurea‐associated severe hypoglycemia was the end point. There was a large increased risk of severe hypoglycemia associated with *CYP2C9* decreased and nonfunctional variants but only in the presence of normal function *POR* (*1*1) (OR 2.81 (1.30–6.09), *P* = 0.009 per number of *CYP2C9* decreased‐function alleles). No effect of decreased function *CYP2C9* alleles was seen in the presence of the *28 allele of POR, which is reported to increase CYP2C9‐mediated activity, thus potentially compensating for decreased function of CYP2C9. Whilst there was no analogous effect on efficacy, assessed by HbA1c reduction, when dose of sulfonylurea was taken into account in a composite endpoint, the decreased function *CYP2C9* alleles were associated with greater efficacy but only on the POR*1*1 background. This study highlights the complexity of pharmacogenetic studies and how analyzing one gene in isolation can be overly simplistic—e.g., the impact of CYP variants will be likely to rely on appropriate transport of sulfonylurea into the liver; and impact of pharmacodynamic variants such as *TCF7L2* or the *KCNJ11/ABCC8* variants may be masked if CYP activity is not taken into account.

Finally, despite being widely used, unlike metformin there has not yet been GWASs reported for sulfonylureas. These could shed novel insight into the PK and PD of sulfonylureas and are eagerly awaited.

## Thiazolidinedione Efficacy

Thiazolidinediones (TZDs), often referred to as glitazones, are PPARγ agonists. They are potent insulin‐sensitizing drugs that primarily work on adipose to promote differentiation from pre‐adipocytes to adipocytes. Pharmacogenetic studies are limited and have focused on PK of the TZDs and variation in the key target gene PPARγ.

As for sulfonylureas, the largest effects are seen for variants altering PK of the TZDs. Hepatic uptake of TZDs is mediated by OAT1B1 with metabolism mostly by CYP2C8. These were evaluated together in a moderate‐sized observational study, again in the GoDARTS cohort;^44^ 833 patients were identified who started TZD, of whom 273 were treated with pioglitazone and 519 with rosiglitazone. For those treated with rosiglitazone, those carrying the increased function CYP2C8*3 allele had less HbA1c reduction than wild type (allelic β = −0.21%, *P* = 0.01) and experienced less weight gain (allelic β = −0.93 kg, *P* = 0.02). The *SLCO1B1 521C* (rs4149056) variant was associated with greater HbA1c reduction (allelic β = 0.18%, *P* = 0.04), but not weight gain, after rosiglitazone treatment.^44^ When the two variants were considered together, the patients who had reduced transport of OAT1B1 and normal metabolism of CYP2C8 had a 0.39% (4 mmol/mol; *P* = 0.006) greater HbA1c reduction than the poor responders.^44^ Interestingly, no effect of these variants was seen with pioglitazone—this is likely to reflect the fact that, in contrast to rosiglitazone, pioglitazone metabolites are active, so *CYP2C8* variants would not be predicted to alter response.

The *Pro12Ala* (rs1801282) variant in *PPARγ* was one of the first robustly replicated type 2 diabetes risk variants. Given that this is the target gene of thiazolidinediones, it is a strong candidate for altered glycemic response to TZDs. However, limited studies have reported on this variant. The most robust study reported is still small, examining TZD response in 250 Chinese patients. Compared with wild‐type individuals, the 104 carriers of the minor allele had an OR 2.32 ((95% CI = 1.10–4.87) *P* = 0.03) of being a responder.^45^ Association of the same variant with a linear reduction in HbA1c and fasting plasma glucose after pioglitazone therapy was replicated in an independent cohort of 67 patients.^46^ A similar trend was reported in 198 Korean patients treated with 4 mg rosiglitazone daily for 3 months.^47^

## DPP‐4 Inhibitor Efficacy

The only robust study for the DPP‐4 inhibitors identified that a variant (rs7202877) near the chymotrypsinogen B1/2 (*CTRB1/2*) gene was associated with glycemic response to DPP‐4 inhibitors. Here the initial signal identified the genetic variant to be associated with glucagon‐like peptide‐1–induced insulin secretion. *CTRB1/2* encodes chymotrypsin, and the rs7202877G allele was associated with an increased fecal chymotrypsin activity. A follow‐up investigation identified the same variant to be associated with HbA1c reduction in 49 patients from the Netherlands and 305 patients from GoDARTS: Carriers of the rs7202877G allele showed 0.51 ± 0.16% lower HbA1c reduction compared with the rs7202877 TT genotype (*P* = 0.0015) after being on gliptins for at least 3 months.^48^

## Future Studies of Pharmacogenetics in Diabetes

Like the rest of the genetics community, there is increasing international collaboration resulting in increasing numbers of patient data available for pharmacogenetic studies. To date, these have been largely based upon observational studies based upon record linkage, but pharmaceutical companies are increasingly making their trial data available and are more routinely incorporating genetic studies into their trial protocols. This will result in increasing opportunity—not only for discovery of genetic variants associated with response but also to evaluate drug side effects (e.g., nausea with GLP1RA or edema with thiazolidinediones) and other nonglycemic outcomes such as cardiovascular risk. Another emerging area where we may potentially see larger effect sizes for drug efficacy in diabetes comes from the increasing use of polygenic risk scores. There are now 400 variants for type 2 diabetes now identified, which when summed as a polygenic risk score can explain large differences in risk.^49^ For example, the top and bottom 2.5% of the distribution differ in risk of diabetes by ~ 10‐fold. These 400 variants can be grouped together according to the pathophysiological process they affect into what have been termed process‐specific polygenic risk scores, e.g., those affecting beta‐cell function, or fat distribution.^49^, ^50^ These have modest effects on the underlying trait, and as diabetes drugs act on these particular traits, one would hope that these process‐specific polygenic risk scores will have moderate impact on glycemic response to diabetes drugs.

## Conclusions

**Figure 4:**
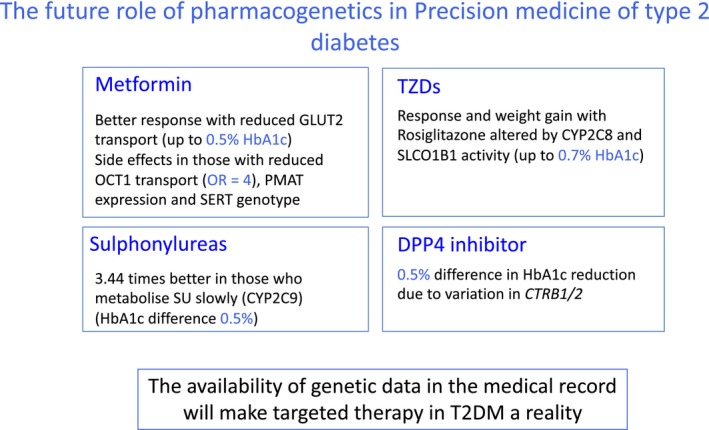
The future role of pharmacogenetics in type 2 diabetes—a summary of the robust and clinically relevant drug–gene interactions that will be of likely clinical utility but only when a preemptive genotyping approach is adopted. CYP, cytochrome P450; DPP‐4, dipeptidyl peptidase‐4; HbA1c, hemoglobin A1c; OR, odds ratio; OCT1, organic cation transporter 1; PMAT, plasma membrane monoamine transporter; SU, sulfonylurea; T2DM, type 2 diabetes mellitus; TZDs, thiazolidinediones.

In current clinical practice, genetic etiology of diabetes has been shown to have a big impact on treatment response in the small group of patients who have monogenic diabetes, and for these patients finding the genetic etiology can be life‐changing—transitioning successfully off insulin onto sulfonylurea treatment. However, for common complex diseases such as type 2 diabetes, it is unlikely that large‐effect etiological variants will be identified due to the genetic architecture of these diseases being driven largely by multiple small effect variants. Whilst a number of robust genetic variants have been established to be associated with glycemic response to metformin, sulfonylureas, thiazolidinediones, and DPP‐4 inhibitors, summarized in **Figure** **4**, these effects are not large enough to justify genetic testing before deciding on treatment—the delay, cost, and limited predictive utility make this impractical and not cost‐effective. However, with the costs of genotyping platforms now as low as £30, a threshold has been reached where preemptive genotyping can (and should) be undertaken, with results embedded in the medical record. At this point, if the choice of second‐line or third‐line therapies is at equipoise, and the genetic information is already available, then these genetic factors can be used to aid in prescribing choice. In order for use of preemptive genotyping for all to become reality, focus now needs to be on processes to facilitate this. For example, mapping from genotype to clinical recommendation will be a dynamic process, as novel pharmacogenetic findings are identified; given this, it will probably be best to hold the genetic and mapping information centrally (within the National Health Service or healthcare provider) to be queried at the point of prescribing by primary or secondary care clinical decision support tools. However, to answer the question posed in the title of the review, I am in no doubt that pharmacogenetics will become mainstream in the management of diabetes—not only monogenic diabetes but also in type 2 diabetes.

## Funding

E.R.P holds a Wellcome Trust Investigator Award 102820/Z/13/Z.

## Conflict of Interest

E.R.P has received honoraria from Lilly, MSD, and Novo Nordisk.
